# A Painless Application of Arsenical Paste

**Published:** 1900-01

**Authors:** William Perrin Nicolson

**Affiliations:** Atlanta, Ga.


### A PAINLESS APPLICATION OF ARSENICAL PASTE.

By WILLIAM PERRIN NICOLSON, M.D.,

ATLANTA, GA.

The application of arsenious acid paste for the removal of malignant growths has been much restricted by the severe resulting pain produced during its escharotic action, so that its use has been far less frequently employed than its effects have warranted. The combinations with morphia and cocain have been of slight material assistance in controlling the pain—the former on account of weak local action as an anesthetic, and the later because of the temporary nature of the anesthesia. The following case illustrates how satisfactorily this result was obtained by the use of orthoform.

White male, aged sixty, presented with a growth upon the right side of the head, projecting outward about to the length and size of a large hen's egg. The base of the tumor extended from just at the outer edge of the orbit to the other a short distance in front of the ear. The surface was covered with a dried scab, from under which blood oozed at the slightest touch. As the tissue seemed very friable a somewhat novel means of removal was employed. A thread of silkworm-gut was passed around the base, and by a rapid sawing motion the growth was quickly removed flush with the skin. After controlling the severe bleeding by pressure applied a short time, the raw surface was freely sprinkled with powdered orthoform. Then with a powder made of equal parts of arsenious acid and powdered gum acacia, orthoform was mixed without noting the proportions. This was then made into a paste and applied to the cut surface and the patient given a few tablets of morphine to take when the pain became severe. The next day we returned and found the tablets unused, the patient having had no pain once the paste was applied. The result was perfect, the growth being entirely destroyed, and to this time, six months after its removal, he remains well.

Though the use of orthoform for the purpose of producing a prolonged local anesthesia when applied to ulcerated or freshly cut surfaces has been well known, I have been unable to find any account of its employment in this manner. Subsequent to the use of it in this case I found the report of a suggestion of its use in weak arsenical solution for application to malignant growths, but in this the arsenious acid was employed only in the strength of one grain to the ounce. As the anesthetic influence of this drug lasts for many hours, it appears to be the ideal combination for such a purpose, and the case reported would seem to open up a wide field of usefulness for orthoform.

